# Utility of preclinical models of altered maternal nutrition to support the developmental origins of health and disease hypothesis

**DOI:** 10.1042/CS20211175

**Published:** 2022-05-16

**Authors:** Mark Hedley Vickers

**Affiliations:** Liggins Institute, University of Auckland, Auckland, New Zealand

**Keywords:** animal models, developmental programming, fetal origins of disease

## Abstract

A clear link has been established between alterations in the early life environment and the risk for developing a range of cardiometabolic diseases in later life, a process preferentially termed developmental programming. In particular, alterations in the maternal nutritional environment have been associated with a range of adverse health outcomes in offspring across the lifecourse; effects that can be passed on to future generations. Following from the early epidemiological observations that provided the basis for the developmental origins of health and disease (DOHaD) hypothesis, a range of animal models were developed to examine the impact of early life programming and provide empirical data to support the emerging framework. These models became key tools to aid in our understanding of developmental programming as allowed investigation of potential mechanisms, strategies for intervention and transgenerational effects. The study published by Langley and Evans (Clin. Sci. 1994;86(2):217–222; DOI:10.1042/CS0860217), using a rat model of maternal low protein exposure, was one of the first to highlight the impact of an altered maternal nutritional environment on programming of elevated blood pressure in offspring. This work became a hallmark study in the DOHaD field by demonstrating key proof of principle to support the early epidemiological associations and characterizing a key preclinical model that has contributed greatly to our understanding of mechanisms underpinning developmental programming—particularly in the area of cardiovascular and renal function.

Over three decades ago, the seminal work by David Barker and colleagues highlighted an association between early life environmental factors and the risk of developing a range of diseases in later life including cardiovascular disease and type 2 diabetes [1]. These observations shaped the fetal origins of adult disease (FOAD) hypothesis and subsequently the developmental origins of health and disease (DOHaD) framework whereby adverse events in early life are shown to impact health across the lifecourse via the process now preferentially termed ‘developmental programming’. Programming pertains to the idea that ‘a stimulus or insult during critical early windows of development may result in long-lasting or persistent effects on the structure or function of an organism’. The early life period represents a window of developmental plasticity and suboptimal prenatal exposures can therefore trigger adaptations that improve fetal survival and allow the developing fetus to prepare for an expected postnatal environment based on maternal cues. However, these adaptations, or predictive adaptive responses (PARs), commonly linked to development of a ‘thrifty phenotype’ [2], may be beneficial for short term survival but may later be disadvantageous when the pre and postnatal environments are mismatched [3]. In particular, alterations in the maternal nutritional environment are now well-recognized as critical factors in shaping the life-long health of the next generation and beyond. One of the most cited observations in humans to date is that of the Dutch Famine (Dutch Hunger Winter) where famine exposure was associated with a range of cardiometabolic outcomes in offspring, effects that were passed to future generations [4]. To provide empirical evidence to support the emerging epidemiological observations, experimental animal models across a range of species were developed that spanned a range of nutritional challenges across different key developmental windows. These models have become key tools to aid our understanding of developmental programming, the mechanisms involved, potential strategies for intervention and transgenerational impacts.

The early work by Barker and colleagues in the Hertfordshire cohort linked being born of low birth weight to an increased risk of death due to ischemic heart disease [5]. Early animal models therefore predominantly focused on maternal nutritional restriction to induce growth restriction to mimic the epidemiological data that formed the basis of the original FOAD hypothesis. In particular, a number of rodent models were developed to induced fetal growth restriction and typically used one of two dietary approaches; global maternal undernutrition or a maternal low protein (LP) diet [6]. Rodent models offer some advantages over other models including small size and relative low cost, ease of maintenance, short life cycle, ease of genetic modification and opportunities for investigating transgenerational programming impacts.

The original paper by Langley and Jackson, using a rat maternal LP model, was one of the first to show the effects of an adverse early life nutritional environment on programming of blood pressure [7]. In this work, female rats were fed either a maternal control diet (18% protein) or habituated to a graded series of LP diets (12, 9 and 6% by weight) for 2 weeks and then throughout pregnancy. After birth, mothers and offspring were fed a standard control diet (20% protein) throughout the period of lactation. The maternal LP diets resulted in a graded phenotypic response with effects being more pronounced in those offspring exposed to the 9 and 6% diets. Systolic blood pressure (SBP) was measured in female offspring at 9 weeks of age using tail cuff plethysmography. Offspring from the 12, 9 and 6% LP groups demonstrated significantly elevated SBP as compared with the control group (18% protein). The study also demonstrated an inverse relationship between maternal protein intake and offspring SBP. In the 9 and 6% protein fed mothers, the elevations in SBP in offspring were still present at 21 weeks of age. The increases in SBP were associated with increased pulmonary angiotensin-converting enzyme activity in the LP-exposed groups. These original observations were therefore consistent with the hypothesis that exposure to a suboptimal diet *in utero* can ‘irreversibly impair aspects of physiological and biochemical function in the fetus’ with adverse impacts on offspring health across the lifecourse.

At the time of the original publication, animal models were still gaining momentum in the DOHaD space and the DOHaD hypothesis itself was still the target of some scepticism (although is now widely acknowledged as a true developmental phenomenon with increasing recognition that a lifecourse approach is now required to reduce noncommunicable disease risk). This is reflected in some of the original comments made on the manuscript around experimental limitations [vol. 86(2), pg 121) albeit acknowledging that the work, if confirmed, could influence public health measures. It was noted that although the work suggested ‘that maternal protein restriction may be yet another means of producing experimental hypertension in rats, it must be considered inconclusive’. Thus, the developmental programming implications of the original work appeared underestimated and although some of the initial comments raised were largely beyond the scope of the original study, it helped pave the way for considerations made for future key studies in this area. In this regard, the points raised served to further progress the field and remain relevant today [8], including the need to examine sex-specific effects, consideration of experimental power and examining the potential differential effects of the timing and duration of the nutritional insult. Indeed, a follow-on paper later the same year by the authors confirmed the original results and further went on to demonstrate that the effects of maternal LP diet exposure on SBP were present in both male and female offspring and were independent of changes in maternal blood pressure [9]. The original model used by Langley and Jackson was also key in further elucidating of potential mechanisms involved in the programming of hypertension in offspring as a result of renal nephron deficit in the setting of a maternal LP diet [10]. Moreover, the graded maternal LP diet approach utilized in this paper also importantly served to inform future studies by those in the developmental programming research field as to the level of protein deficiency required to optimize the LP model (typically 8–9%). Of note, the LP diet itself does not increase blood pressure in adult rats thus reinforcing that the effects observed reflect a direct developmental programming phenomenon. It also needs to be recognised that, although the original work by Barker et al. focused on low birth weight as a proxy for later programming, the work by Langley and Evans also highlighted that adverse programming effects can occur in the absence of marked changes in fetal weight or postnatal growth trajectories.

As such, this work represents one of the key hallmark studies in the DOHaD domain with the results replicated across several studies and paving the way for extended studies examining underlying mechanisms and potential transgenerational impacts. Importantly, since this early work by Langley and Jackson, the variety of small animal models used in the DOHaD field have consistently produced phenotypes that display some form of aberrant metabolic phenotype that reflect alterations in the early life environment and have served to further confirm and reinforce the programming paradigm. There do, however, exist some differences across the cardiometabolic phenotypes in the setting of the LP model and these likely relate to the different strains used, severity of the LP restriction and the LP dietary protocols utilised [11]. Indeed, Langley-Evans noted in subsequent work that differing LP dietary manipulations across the rat pregnancy models utilised (e.g. total fat content, fatty acid profiles, methionine content and the source of carbohydrate) can elicit differential programming effects upon the developing cardiovascular system [12]. There also needs to be consideration given to the windows of LP exposure. The Langley and Jackson paper examined gestational LP exposure with a return to a standard control diet during lactation. Given the potential around direct lactational programming, more work is required to examine the differential effects across pregnancy alone, lactation alone and both pregnancy and lactation on offspring outcomes. This was highlighted in work by Howie et al. in the rat whereby maternal undernutrition during different critical windows of development resulted in differential and sex-specific effects on postnatal adiposity and related metabolic profiles in adult rat offspring [13]. This also links to an acknowledged limitation of rodent models as regards potential clinical implications of the research findings. As altricial species, the timing of most key developmental processes in rodents, including wiring of neuronal circuitry, occurs in the first 2 weeks of postnatal life as compared with late gestation in humans and as shown for other large animal models of programming. Further, there also needs to be mention of the approach used to assess SBP. Although the tail-cuff method has been widely used in rodents and validated against other measurement methods [14], there is some evidence that the programmed increases in SBP in rodent models may reflect a differential stress response to the measurement environment itself rather than persisting effects on SBP. Work by O’Regan et al., using a model of prenatal dexamethasone exposure, measured blood pressure using telemetry and reported lower basal blood pressures in male and female adult offspring [15]. This contrasted with work by others using the tail-cuff method in this and similar programming models but since direct comparisons across the two measurement methods were not made in the same cohort of animals and this was a dexamethasone-based model, further work is required to confirm these observations in the context of nutritional programming. Nonetheless, the data to date further emphasises the role of alterations in critical early life developmental windows in establishing disease risk and the need for a lifecourse strategy to mitigate common diseases rather than treating once already manifest. In this context, although early work in the DOHaD domain considered developmental programming to result in an irreversible change in developmental trajectory, evidence to date, at least from animal models, suggests that programming may be ameliorated or even reversed via targeted early life interventions [16].

The original work by Langley and Jackson provided key proof of principle to support the epidemiological associations and provided a key experimental platform of which has contributed greatly to our understanding of DOHaD mechanisms, particularly in the area of cardiovascular and renal programming. Further, given that developmental programming can occur at both ends of the maternal nutritional spectrum i.e. the ‘U-shaped’ response curve, this work also helped pave the way for the development of models of maternal overnutrition which are becoming increasingly relevant in the setting of DOHaD. The originality and ongoing relevance of this work is reflected in over 600 citations and helped establish both the importance and relevance of small animal models in the DOHaD domain and reflects an experimental approach that is still used widely today as a key and robust tool to aid in our understanding of the pathogenesis of early life programming of cardiometabolic disease.

## Open Access

Open access for this article was enabled by the participation of The University of Auckland in an all-inclusive *Read & Publish* agreement with Portland Press and the Biochemical Society under a transformative agreement with CAUL.

## Abbreviations

DOHaD: developmental origins of health and disease
FOAD: fetal origins of adult disease
LP: low protein
PAR: predictive adaptive response
SBP: systolic blood pressure
